# Comment on ‘Impact of Resistance Training and Chicken Intake on Vascular and Muscle Health in Elderly Women’ by Fujie et al.

**DOI:** 10.1002/jcsm.13803

**Published:** 2025-04-14

**Authors:** Pincheng Luo, Yihan Shi, Yanxue Lian

**Affiliations:** ^1^ School of Medicine University of Galway Galway Ireland; ^2^ School of Medicine Newcastle University Newcastle upon Tyne UK

Dear Editor,

We read with great interest the recent article by Fujie et al. [1] published in your esteemed journal. The study provides valuable insights into the effects of moderate‐to‐high‐intensity resistance training (RT) combined with high‐protein intake (steamed chicken breast) on arterial stiffness, muscle mass, strength and quality in elderly women. However, we would like to highlight several limitations and areas for future research that could enhance its impact.

First, the study lacks a personalized training approach tailored to individual participants. The study employed a standardized resistance training protocol, adjusting weights every 2 weeks based on one‐repetition maximum (1‐RM) measurements. While this approach ensures consistency, it does not account for individual variations in baseline fitness, recovery capacity or adaptive response to training. Although the authors note no significant differences in one‐repetition maximum (1‐RM) of leg extension or leg curl at baseline RT and resistance training plus higher dietary animal protein intake (RT + HP) groups, this does not negate the need for personalized programming. Research has shown that tailoring exercise regimens by modifying intensity, frequency and exercise selection can lead to greater improvements in muscle function and cardiovascular health [2]. Future studies could benefit from incorporating personalized training plans to optimize individual outcomes.

Second, the study focused primarily on lower limb exercises, overlooking other muscle groups essential for functional independence and overall health. Resistance training programmes for elderly individuals should ideally include exercises targeting the upper body, core and postural muscles to address the multifactorial nature of age‐related decline. A more comprehensive approach to exercise programming, incorporating a wider range of exercises tailored to individual needs and functional goals, could enhance the study's applicability and outcomes.

Finally, the authors propose that the attenuation of arterial stiffness in the resistance training plus higher dietary animal protein intake (RT + HP) group may be due to angiotensin‐converting enzyme (ACE) inhibition by high‐protein intake. While this is a plausible hypothesis, it remains speculative without direct evidence. Although baseline data showed no significant differences in circulating angiotensin II (Ang II) levels among the groups, this alone is insufficient to confirm the proposed mechanism. Measuring ACE activity or expression in vascular tissues, along with circulating Ang II levels, could provide more conclusive insights into this potential pathway.

In conclusion, while the study offers valuable contributions to the field, addressing these limitations in future research could further elucidate the mechanisms underlying the observed benefits and optimize interventions for improving vascular and muscle health in elderly women.

We sincerely appreciate the contributions made by the authors to this research and hope the authors can consider these points in a reply letter and in their future research.

## Disclosure

Yanxue Lian is the guarantor of this study and accepts full responsibility for its integrity and accuracy.

## Ethics Statement

The authors have nothing to report.

## Conflicts of Interest

The authors declare no conflicts of interest.

## Data Availability

Data sharing is not applicable to this article as no new data were created or analysed in this study.
